# Supplementary material for: Pillar arrays as tunable interfacial barriers for microphysiological systems
Source: Commun Eng. 2025 Nov 20;4:197. doi: 10.1038/s44172-025-00527-x (PMC12634667; doi:10.1038/s44172-025-00527-x)
Supplement: Supplementary file 2 — Supplementary Information [file 44172_2025_527_MOESM2_ESM.pdf]

Supplementary Figures

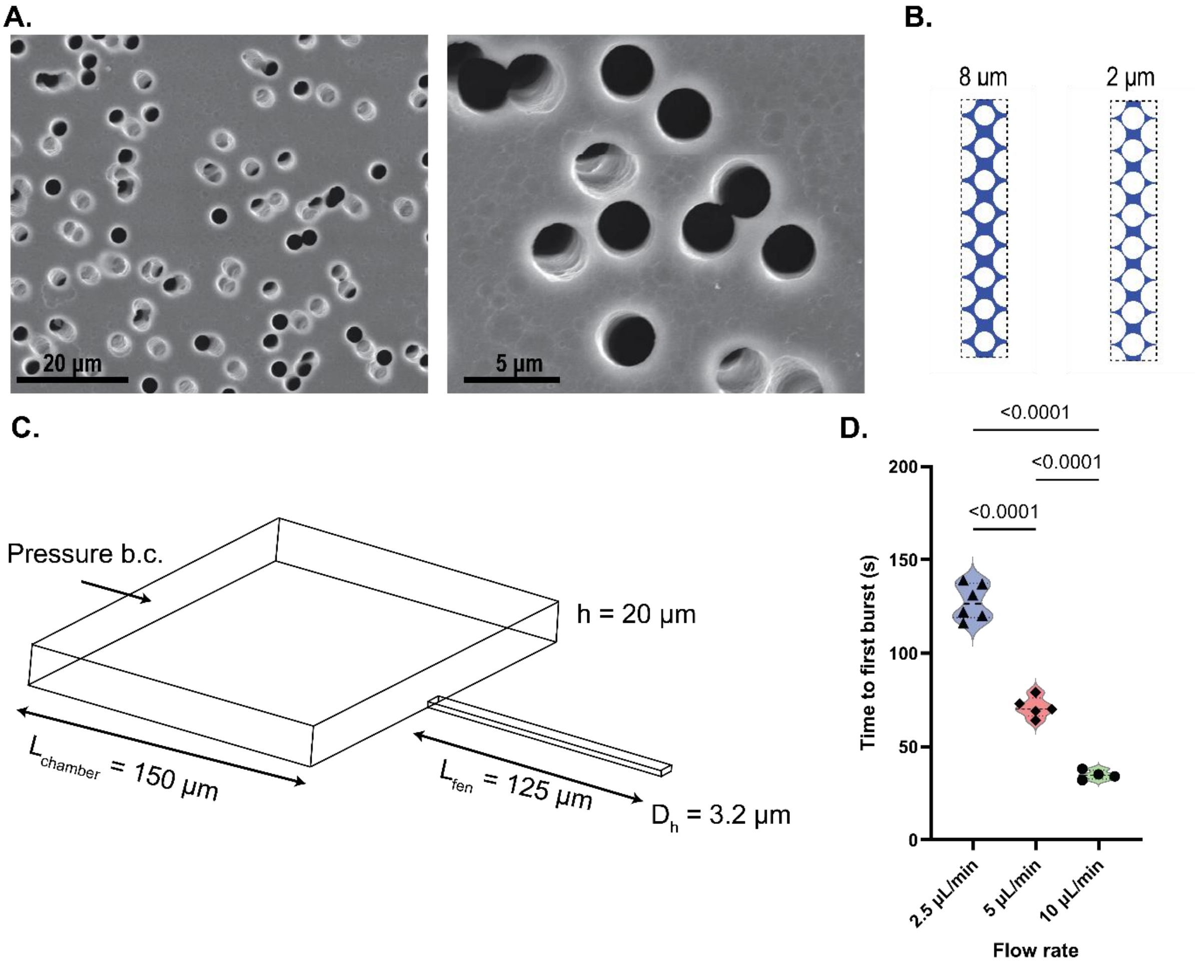

Supplementary Figure 1: Characterization and modeling of MPS interfacial barriers (A) SEM images of commercially available PET membrane showing pores that overlap forming unwanted larger openings, inter-pore spacing, and pore densities. **(B)** Variation of pore size as defined by the distance between the pillars changes the porosity of the pillar-based fenestration layer. Shown here are two interfaces created in a 125 µm x 708 µm rectangle with 8 µm and 2 µm pore sizes and porosity of 31% and 19%, respectively. **(C)** A single microchannel-based fenestra finite element model was used to simulate the movement of the air-water interface across the microfluidic barrier to quantify burst pressure. (**D**) The time to first burst, used to quantify the barrier function, with FITC-doped cell culture medium at three different flow rates.

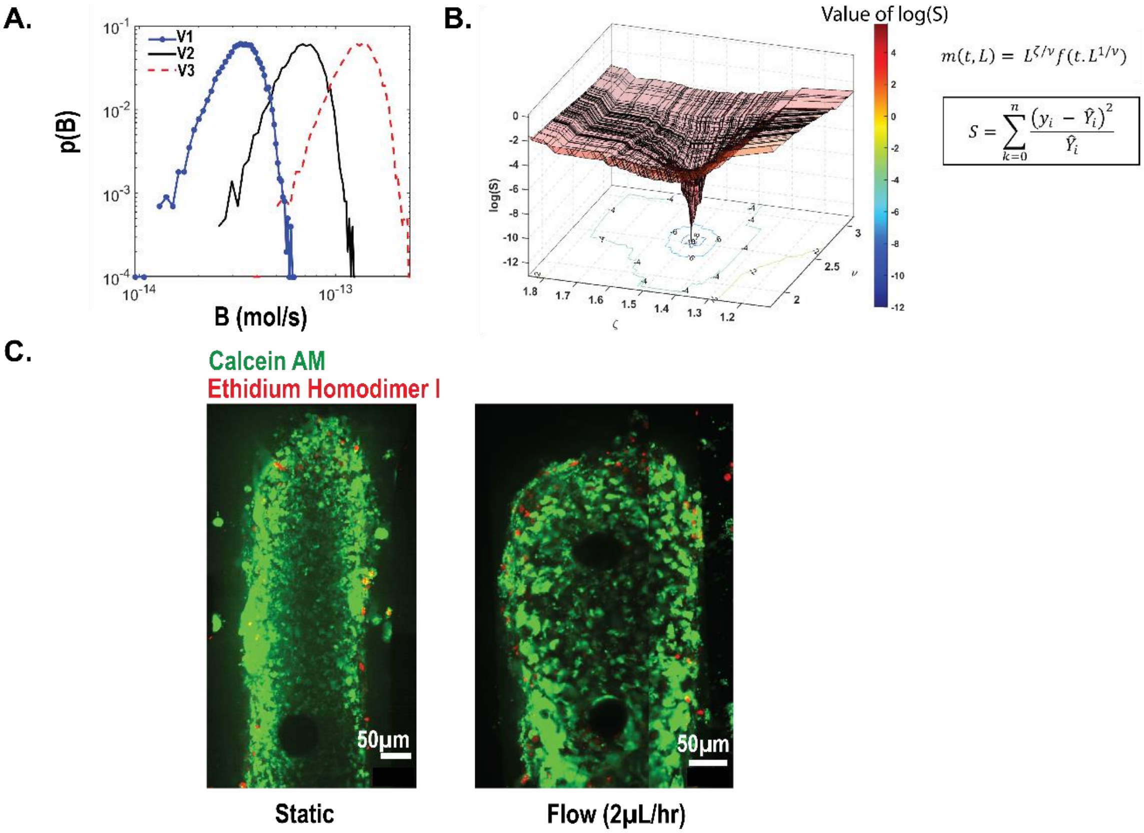

Supplementary Figure 2: Supporting data for finite size scaling of OCR data and experimental measurement of viability to validate computational predictions. (A) Tissue OCR B probability distributions obtained for V1, V2, and V3 using FEM PoM method. **(B)** Differential evolution approach was used to obtain parameters for probability collapse. Shown here is the estimation of parameters ν and ζ for the collapse of probability distributions of m to estimate the scaling with respect to L and t, described by a scaling function f. **(C)** Representative images of calcein AM and ethidium homodimer I staining to evaluate live and dead cells within tissues cultured at static and under perfusion of 2 µL/hr. Images shown are in the middle of the tissue height.
