# Supplementary material for: Pillar arrays as tunable interfacial barriers for microphysiological systems
Source: Commun Eng. 2025 Nov 20;4:197. doi: 10.1038/s44172-025-00527-x (PMC12634667; doi:10.1038/s44172-025-00527-x)
Supplement: Supplementary file 3 — Description of Additional Supplementary Files [file 44172_2025_527_MOESM3_ESM.pdf]

Description of Additional Supplementary Files

File name: Supplementary Movie 1

Description: A fluctuation of the liquid advancing front in the capillary valve
